# Prevalencia de anomalías coronarias detectadas por tomografía computarizada en el Instituto Nacional Cardiovascular - INCOR

**DOI:** 10.47487/apcyccv.v3i3.233

**Published:** 2022-09-30

**Authors:** Zoila I. Rodríguez Urteaga, Luis E. Murillo Pérez, Aurelio Mendoza Paulini, Luisa S. Talledo Paredes

**Affiliations:** 1 . Cardiología no invasiva. Instituto Nacional Cardiovascular “Carlos Alberto Peschiera Carrillo” - INCOR. Lima, Perú. Cardiología no invasiva Instituto Nacional Cardiovascular “Carlos Alberto Peschiera Carrillo” - INCOR Lima Perú

**Keywords:** Arterias Coronarias, Angiografía por Tomografía Computarizada, Malformaciones de los Vasos Coronarios, Perú, Coronary Vessels, Computed Tomography Angiography, Coronary Vessel Anomalies, Peru

## Abstract

**Objetivo:**

determinar la prevalencia de anomalías coronarias (AC) en pacientes evaluados por tomografía computarizada (TC) de 64 detectores en el Instituto Nacional Cardiovascular en el Perú entre los años 2016 a 2020.

**Materiales y métodos:**

estudio observacional retrospectivo, en el cual se revisaron las TC de arterias coronarias de 1486 pacientes, realizadas en un tomógrafo de 64 filas de detectores, en busca de anomalías coronarias.

**Resultados:**

la prevalencia de AC detectada por TC fue de 4,71% (70 casos) de ellos 64,3% varones. Las anomalías de origen fueron las más frecuentes, de ellas el nacimiento de una arteria coronaria desde el seno coronariano opuesto fue la más común (48,6%), siendo la coronaria derecha la principal arteria anómala (31%), y el principal trayecto fue el interarterial (31%). El origen anómalo del tronco coronario izquierdo desde la arteria pulmonar se encontró en cinco pacientes. Entre las anomalías de la anatomía arterial coronaria intrínseca, la principal fue la doble arteria descendente anterior (10%). Las fístulas coronarias representaron el 11,4% de casos.

**Conclusiones:**

la prevalencia de AC detectadas por TC de 64 detectores en un instituto del Perú fue de 4,71%. La principal anomalía coronaria fue el origen de la arteria coronaria derecha desde el seno coronariano izquierdo con trayecto interarterial.

## Abstract (en)

**Objective:**

: To determine the prevalence of coronary anomalies (CA) in patients evaluated by 64-detector computed tomography (CT) at the Instituto Nacional Cardiovascular in Peru between 2016 and 2020.

**Materials and methods:**

Retrospective observational study, coronary artery CT scans of 1486 patients were performed on a 64-detector row CT scanner and reviewed in search for coronary anomalies.

**Results:**

The prevalence of CA detected by CT was 4.71% (70 cases) of which 64.3% were male. Abnormalities of origin were the most frequent, of which the origin of a coronary artery from the opposite coronary sinus was the most common (48.6%), with the right coronary being the main anomalous artery (31%), and the main path was interarterial (31%). Anomalous origin of the left main coronary from the pulmonary artery was found in 5 patients. Among the anomalies of the intrinsic coronary arterial anatomy the most frequent was the double left anterior descending artery (10%). Coronary fistulas accounted for 11.4% of cases.

**Conclusions:**

The prevalence of CA detected by 64-detector CT in a Peruvian institute was 4.71%. The most frequent coronary anomaly was the origin of the right coronary artery from the left coronary sinus with interarterial trajectory.

## INTRODUCCIÓN

Las anomalías coronarias se definen como una característica morfológica inhabitual que se presenta en menos del 1% de la población en general, a diferencia de una variante anatómica, la cual supera este porcentaje ^1^^-^^3^. La prevalencia de las anomalías coronarias varía ampliamente en la literatura y depende de la definición utilizada y del método empleado para su evaluación; así, oscila entre el 0,3 - 5,6% en pacientes sometidos a angiografía coronaria invasiva y es aproximadamente el 1% de las autopsias de rutina ^3^.

El método diagnóstico utilizado con mayor frecuencia era la angiografía coronaria invasiva (ACI); sin embargo, la mayor radiación y las imágenes bidimensionales brindadas podrían llevar a una interpretación inexacta de los resultados en algunos casos. La verdadera prevalencia de anomalías coronarias en la población general puede haber sido subestimada en base a la ACI, pues esta prevalencia es sustancialmente más alta con tomografía computarizada (TC) que con ACI ^4^. Por consiguiente, el procedimiento actual más empleado es la TC, ya que permite una visualización tridimensional y una mejor resolución espacial, así como la delimitación del trayecto, incluyendo el inicio, curso y final de las arterias coronarias, evaluación del estrechamiento luminal anómalo de las arterias coronarias, la relación con las estructuras circundantes y la enfermedad de las arterias coronarias ^5^^-^^8^.

Se desconoce la frecuencia y las características de las anomalías coronarias en nuestra realidad, por lo que el objetivo general del estudio fue determinar la prevalencia de anomalías coronarias en pacientes evaluados por TC en el Instituto Nacional Cardiovascular INCOR, en el Perú.

## MATERIALES Y MÉTODOS

### Diseño de estudio

Se realizó un estudio descriptivo, observacional, retrospectivo en el Instituto Nacional Cardiovascular «Carlos Alberto Peschiera Carrillo» - INCOR, el cual es un instituto de referencia nacional en patología cardiovascular perteneciente al Seguro Social de Salud del Perú. Se recolectó la información de un periodo de 5 años comprendido entre el 2016 al 2020. 

### Población de estudio

Los criterios de inclusión fueron todos los pacientes que se hayan realizado TC para evaluación de arterias coronarias, sin límite de edad. Se excluyó a pacientes con estudios de TC con mala calidad de imagen, con estudios que no se encontraron en el sistema RIS PACS (*Picture Archiving and Communication System*) y con cardiopatías congénitas complejas asociadas (tetralogía de Fallot, transposición de grandes arterias, doble vía de salida ventricular, truncus arterioso y corazón univentricular).

### Variables de estudio

Se obtuvieron datos de las principales características de las anomalías coronarias (origen, trayecto y/o terminación anómalos). En la revisión de la base de datos de tomografía, se recolectó información de edad, sexo y la descripción del informe tomográfico. 

### Procedimientos

Cada estudio de TC de arterias coronarias fue analizado por un médico cardiólogo especialista en tomografía computarizada cardiaca y, posteriormente, se revisó el informe de tomografía de cada estudio (localizado en la base de datos de tomografía del Instituto Nacional Cardiovascular «Carlos Alberto Peschiera Carrillo» - INCOR) para corroborar los diagnósticos. En caso de existir discrepancias entre la lectura actual del cardiólogo con el informe tomográfico de la base de datos, un segundo cardiólogo especialista en tomografía cardiaca revisó las imágenes para llegar a un consenso en el diagnóstico.

Los estudios fueron realizados con un tomógrafo Toshiba de 64 filas de detectores, con gatillado prospectivo y retrospectivo, a 80-120 kV, según el peso del paciente. Los pacientes que acudieron a tomografía computarizada de arterias coronarias fueron en su mayoría medicados con betabloqueadores o calcio-antagonistas no dihidropiridínicos por vía oral; además, recibieron nitratos vía sublingual. Mientras que los pacientes que acudieron para estudios de angiotomografía de aorta torácica y de corazón morfológico, no recibieron medicación previa al estudio de tomografía computarizada.

### Aspectos éticos

El presente estudio fue aprobado por el Comité de Ética de Investigación del Instituto Nacional Cardiovascular «Carlos Alberto Peschiera Carrillo» - INCOR (Certificado de Aprobación: 13/2020-CEI). Al ser un estudio observacional retrospectivo no se obtuvo consentimiento informado de los participantes; sin embargo, se realizaron estrategias para mantener la privacidad de la información de estos (base de datos codificada).

### Análisis de datos

Los datos fueron organizados en tablas y gráficas con ayuda del programa SPSS Statistics versión 25.0 para Mac. La edad fue analizada de forma cuantitativa y después fue categorizada por grupos etarios. Las demás variables obtenidas fueron analizadas de forma cualitativa. Se aplicaron medidas de tendencia central como media aritmética, desviación estándar; así como frecuencias absolutas y relativas.

## RESULTADOS

Se revisaron 2018 estudios de TC de arterias coronarias, de aorta torácica y de corazón, realizados en el INCOR entre los años 2016 a 2020; de los cuales, 1486 estudios cumplieron con los criterios de inclusión y exclusión. De estos últimos, 70 consideraron anomalías coronarias, representando el 4,71%. La prevalencia en pacientes sin diagnóstico de la anomalía (por ecocardiografía o ACI) previo al estudio de TC coronaria, fue de 3,2%.

**Figura 1:**
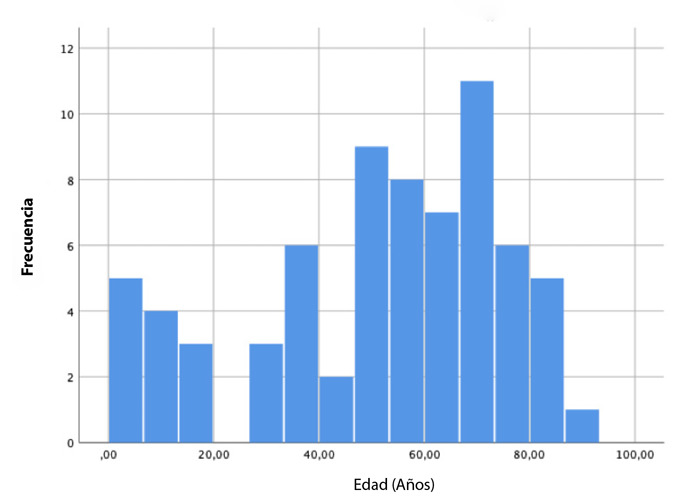
Anomalías coronarias según grupos de edad.

La edad media fue de 51 ± 24,5 años) (Figura 1). La edad mínima fue de 3 meses y la máxima de 90 años. Doce pacientes fueron menores de 18 años. Las anomalías ocurrieron con mayor frecuencia en varones (64,3%) que en mujeres, con una relación de 1,8:1.

Los motivos del estudio de TC fueron: dolor torácico típico (8,6%), dolor torácico atípico (7,1%), disnea (8,6%), palpitaciones (4,3%), síncope (2,9%), alteraciones electrocardiográficas (4,3%), asintomáticos (10%), referidos de ACI (24,3%), referidos de ecocardiografía (7,1%) y como un estudio prequirúrgico o preprocedimiento intervencionista (22,9%).

Clasificamos a las anomalías coronarias en tres grandes grupos: anomalías de origen y trayecto, anomalías de la anatomía arterial coronaria intrínseca y anomalías coronarias de término (Tabla 1). Algunos pacientes presentaron más de un tipo de anomalía coronaria.

**Tabla 1:** Clasificación de las anomalías coronarias por tomografía computarizada en 70 casos encontrados

Anomalía coronaria	n (%)
Anomalías de origen y trayecto	
Origen independiente de DA y CX	4 (5,7)
Origen alto de CD desde aorta ascendente	10 (14,3)
Origen de TCI desde AP	5 (7,1)
Origen de una arteria coronaria desde el seno coronariano opuesto	34 (48,6)
Arteria coronaria única	4 (5,7)
CD única	2 (2,9)
TCI único	2 (2,9)
Anomalías de la anatomía arterial coronaria intrínseca	
Doble arteria coronaria	8 (11.43)
DA	7 (10)
CD	1 (1,43)
Origen ectópico de la primera rama septal desde TCI	1 (1,43)
Anomalías coronarias de término (fístulas coronarias)	8 (11,4)
CX hacia ventrículo derecho	2 (2,9)
DA hacia AP	2 (2,9)
CD hacia AP	1 (1,43)
DA y CD hacia AP	1 (1,43)
CD hacia aurícula derecha	1 (1,43)
CX hacia seno coronario	1 (1,43)

Algunos pacientes presentaron más de una anomalía coronaria**.** TCI: tronco coronario izquierdo, DA: arteria descendente anterior, CX: arteria circunfleja, CD: arteria coronaria derecha, AP: arteria pulmonar

### Anomalías coronarias de origen y trayecto (Figura 2)

En cuanto a las anomalías de origen, la más común fue el origen de una arteria coronaria desde el seno coronariano opuesto (48,6%) con una prevalencia de 2,3% respecto del total de pacientes evaluados. De ellas, la más frecuente fue el origen anómalo de la arteria coronaria derecha (CD) desde el seno coronariano izquierdo (22 pacientes), seguido del origen de la arteria circunfleja (CX) desde el seno coronariano derecho (7 pacientes); finalmente, el origen del tronco coronario izquierdo (TCI) desde el seno coronariano derecho se presentó en cinco pacientes. Las arterias con origen anómalo desde el seno coronariano opuesto presentaron un ostium independiente en 29 pacientes (85,3%), mientras que cinco pacientes (14,7%) compartían un ostium común.

**Figura 2:**
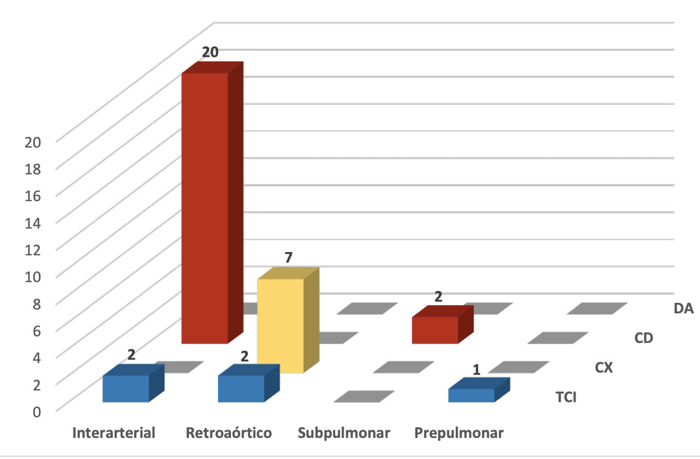
Origen anómalo de arterias coronarias desde el seno coronariano opuesto y su trayecto. TCI: tronco coronario izquierdo, CX: arteria circunfleja, CD: arteria coronaria derecha, DA: arteria descendente anterior.

**Figura 3:**
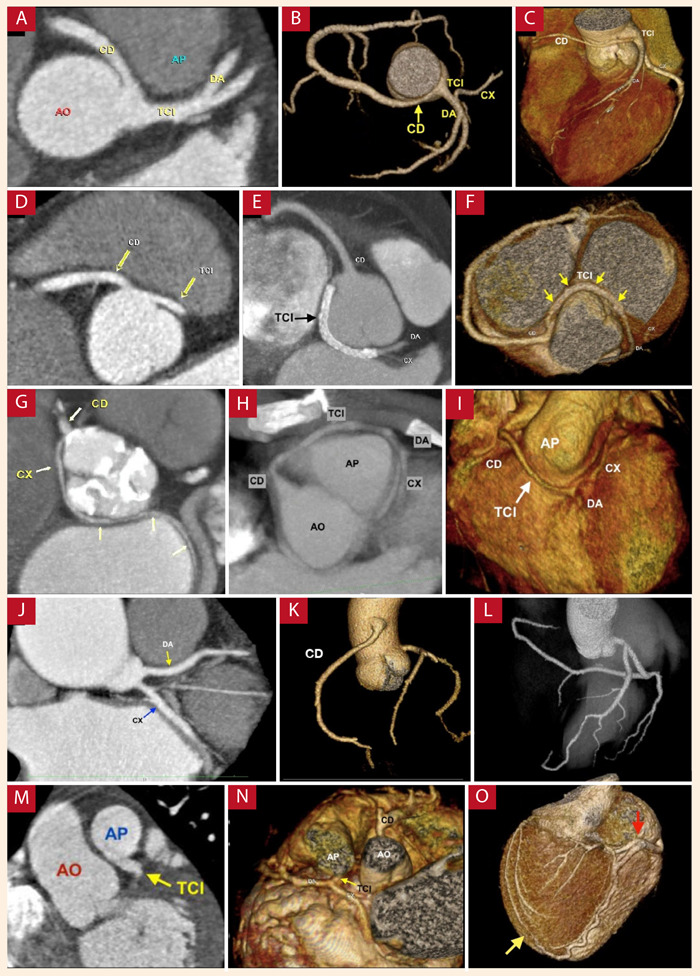
Anomalías coronarias de origen. **A** Origen de la arteria coronaria derecha en el seno coronariano izquierdo con trayecto interarterial. **B y C** reconstrucciones tridimensionales de dos pacientes. **D** Tronco coronario izquierdo con origen en el seno coronariano derecho con trayecto interarterial. **E** Tronco coronario izquierdo con origen en el seno coronariano derecho con trayecto retroaórtico. **F** reconstrucción tridimensional. **G** Arteria circunfleja con origen en el seno coronariano derecho con trayecto retroaórtico. **H** Tronco coronario izquierdo con origen desde la arteria coronaria derecha en el seno coronariano derecho, con trayecto prepulmonar. **I** reconstrucción tridimensional. **J** Origen independiente de las arterias descendente anterior y circunfleja. **K** Arteria coronaria única con agenesia de arteria coronaria derecha, con importante desarrollo de la arteria circunfleja. **L** Origen de la arteria coronaria derecha desde la aorta ascendente. **M** Origen del tronco coronario izquierdo desde la arteria pulmonar. **N** reconstrucción tridimensional. **O** importante desarrollo de la arteria coronaria derecha (flecha roja) que proporciona circulación colateral a ramas diagonales (flecha amarilla). **TCI**: tronco coronario izquierdo, **DA**: arteria descendente anterior, **CX**: arteria circunfleja, **CD**: arteria coronaria derecha, **AO**: aorta, AP: arteria pulmonar.

El trayecto más frecuente en el grupo de anomalías de origen de una arteria coronaria desde el seno coronariano opuesto fue el interarterial (22 pacientes, 64,7%), de los cuales en 20 pacientes (90,9%) se trató de anomalías de origen de la CD (figuras 3A a 3C) y 9,1% del TCI (Figura 3D). Respecto a la presencia de estenosis en el origen de las arterias con nacimiento anómalo desde el seno coronariano opuesto con trayecto interarterial, dos pacientes tuvieron estenosis severa mayor a 70% (desde CD), seis pacientes estenosis moderada de 50 a 69% (desde CD), 13 pacientes estenosis leve menor a 50% (11 desde CD y 2 de TCI), y 1 paciente no presentó estenosis (desde CD).

Las otras anomalías de trayecto halladas fueron: nueve casos retroaórticos (7 de CX y 2 de TCI) (Figuras 3E a 3G), dos casos subpulmonares (desde CD) y 1 prepulmonar (desde TCI) (Figuras 3H y 3I). Las arterias coronarias de origen anómalo desde el seno opuesto cuyo trayecto fue distinto al interarterial, no presentaron estenosis en su origen (once pacientes).

La agenesia del TCI se observó en once pacientes (15,7%), con una prevalencia de 0,74% del total de estudios. De los cuales cuatro pacientes presentaron nacimiento independiente de DA y CX desde el seno coronariano izquierdo (Figura 3J), mientras que siete pacientes presentaron origen de CX desde el seno coronariano derecho (descrito en el párrafo previo) con consiguiente origen directo de DA desde el seno coronariano izquierdo.

El origen de una arteria coronaria desde la aorta ascendente se encontró en diez pacientes (14,3%), con una prevalencia de 0,67% del total de estudios, en todos los casos la CD fue la arteria afectada (Figura 3K). De este grupo, solamente un paciente presentó trayecto interarterial con estenosis moderada de 50 a 69%, el resto no presentaron estenosis.

Se encontró arteria coronaria única en cuatro pacientes (5,7%), con una prevalencia de 0,27% del total de estudios. Según la clasificación de Lipton ^9^, dos fueron anomalías de origen del TCI desde el seno coronariano derecho de un ostium común con CD, uno de ellos con trayecto prepulmonar (Tipo R II - A de Lipton) (Figuras 3H y 3I) y el otro retroaórtico (Tipo R II - P de Lipton). Un paciente con origen de CD desde el seno coronariano izquierdo que se originaba desde el segmento proximal del TCI (Tipo L II - B de Lipton) (Figura 3C). Mientras que la agenesia de CD (Tipo L I de Lipton) (Figura 3L) se encontró solamente en 1 paciente (1,43%), con una prevalencia de 0,07%.

El origen anómalo de una arteria coronaria desde la arteria pulmonar se observó en cinco pacientes (7,1%), con una prevalencia de 0,34% del total de estudios. El TCI fue la arteria anómala hallada en todos los casos (Figuras 3M a 3O). Todos los pacientes con esta anomalía fueron pediátricos (de 3 meses a 7 años) y cuatro de ellos de sexo femenino.

### Anomalías de la anatomía arterial coronaria intrínseca

**Figura 4:**
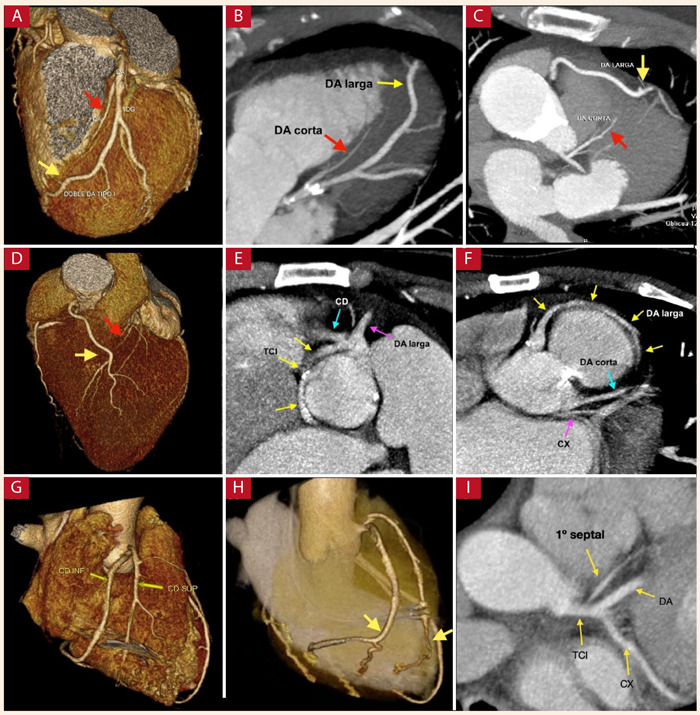
Anomalías de la anatomía arterial coronaria intrínseca. **A y B** Doble arteria descendente anterior tipo I con reconstrucción tridimensional, con arteria descendente anterior corta (flecha roja) y arteria descendente anterior larga (flecha amarilla). **C** Doble arteria descendente anterior tipo IV, arteria descendente anterior corta (flecha roja) y arteria descendente anterior larga (flecha amarilla). **D** reconstrucción tridimensional. **E y F** Doble arteria descendente anterior tipo IV, asociada a arteria coronaria única desde el seno coronariano derecho con TCI con trayecto retroaórtico (Tipo R II - P de Lipton). **G y H** Reconstrucción tridimensional de doble arteria coronaria derecha (flechas). I Origen ectópico de la primera rama septal desde el tronco coronario izquierdo. **TCI:** tronco coronario izquierdo, **DA**: arteria descendente anterior, **CX**: arteria circunfleja, **CD**: arteria coronaria derecha.

Dentro de este grupo de anomalías la más común fue la doble arteria coronaria (ocho pacientes, 11,4%), con una prevalencia de 0,54% del total de estudios evaluados. De estas anomalías, la doble DA fue la más frecuente (siete pacientes). Según la clasificación de Spíndola-Franco ^10^ se encontró cinco pacientes con doble DA tipo I (Figuras 4A y 4B), mientras que dos pacientes presentaron doble DA tipo IV (Figuras 4C a 4F). Solamente se halló un caso (1,43%) de doble CD (Figuras 4G y 4H) que presentó un ostium común.

Otra de las anomalías intrínsecas es el origen ectópico de la primera rama septal, del cual encontramos solamente un paciente (1,43%), en este caso la primera rama septal se originaba desde el TCI (Figura 4I).

### Anomalías coronarias de término

**Figura 5:**
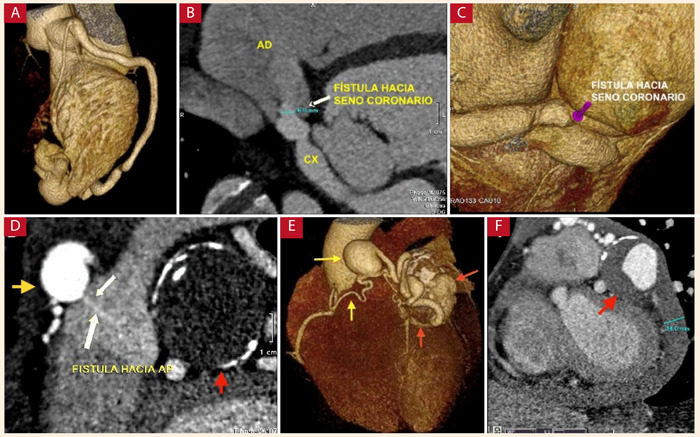
Anomalías de término de las arterias coronarias. **A** Fístula de arteria circunfleja a seno coronario. **B** reconstrucción tridimensional (la flecha señala la desembocadura de la fístula en el seno coronario). **C** Reconstrucción tridimensional de fístula de arteria circunfleja a ventrículo derecho. **D** Aneurisma de fístulas desde la arteria descendente anterior (flecha roja) con trombo mural y desde la arteria coronaria derecha (flecha amarilla) con desembocadura en la arteria pulmonar (flechas blancas). **E** reconstrucción tridimensional. **F** derrame pericárdico por ruptura de aneurisma parcialmente trombosado de fístula de arteria descendente anterior (flecha roja) hacia la arteria pulmonar. **CX**: arteria circunfleja, **AD**: aurícula derecha, **AP**: arteria pulmonar.

Se identificó a ocho pacientes (11,4%) con fístulas coronarias, con una prevalencia de 0,54% respecto total de pacientes evaluados. El origen de la fístula desde las arterias DA, CX y CD fue de tres casos en cada una. El drenaje de las fístulas fue hacia la arteria pulmonar en cuatro pacientes (50%), al ventrículo derecho en dos pacientes (25%) (Figura 5A), a la aurícula derecha en 1 paciente (12,5%) y hacia el seno coronario en 1 paciente (12,5%) (Figuras 5B y 5C). Un paciente presentó dos fístulas desde dos arterias coronarias (DA y CD) hacia la arteria pulmonar, que además se asoció a aneurismas en ambas fístulas y que se complicó con taponamiento cardiaco por ruptura de una de ellas (Figuras 5D a 5F).

## DISCUSIÓN

En el presente estudio se encontró que la prevalencia de anomalías coronarias por TC coronaria fue de 4,7%, siendo la anomalía más frecuente las de origen en el seno coronario contralateral.

La mayoría de las anomalías coronarias congénitas son benignas, pero algunas pueden ocasionar isquemia miocárdica e incluso la muerte cardiaca súbita, tal es el caso del origen anómalo del tronco coronario izquierdo desde la arteria pulmonar, donde estudios de la historia natural de esta anomalía sugieren un mal pronóstico en pacientes no tratados ^7^. En un estudio sobre muerte súbita en 1866 deportistas jóvenes (de 8 a 39 años), las anomalías coronarias, específicamente el origen de una arteria coronaria desde el seno coronariano opuesto, representaron la segunda causa cardiovascular de muerte ^11^.

La alteración del desarrollo coronario durante la embriogénesis da como resultado defectos congénitos coronarios que alteran el flujo sanguíneo de las arterias coronarias ^12^. En base a la fisiopatología se han generado múltiples clasificaciones, dependiendo de las características anatómicas o según su importancia funcional y clínica ^13^. Una de las clasificaciones anatómicas más empleadas es la propuesta por Angelini ^2^^,^^14^, quien considera cuatro grupos: anomalías de origen y trayecto, anomalías de la anatomía arterial coronaria intrínseca, anomalías de la terminación coronaria y vasos anastomóticos anómalos.

La prevalencia reportada de anomalías coronarias por TC varía entre 7,9 a 10,1%, considerando al puente intramiocárdico como una anomalía coronaria ^4^^,^^15^; sin embargo, cuando se lo considera como una variante normal, la prevalencia de anomalías coronarias oscila entre 1,16 a 3,1% ^16^^-^^19^. En este estudio no se consideró al puente intramiocárdico como anomalía coronaria, sino como una variante anatómica.

La prevalencia encontrada (4,71%) fue mayor a lo reportado en otros estudios, y podría deberse a que se incluyó a pacientes pediátricos, a diferencia de otras investigaciones ^16^^,^^18^; además, en algunos estudios no reportaron algunas anomalías como: doble arteria coronaria (DA y CD) y el origen ectópico de la primera rama septal ^18^. Además, la metodología utilizada (a diferencia de otros) consistió en revisar nuevamente todas las TC, y no solamente aquellas que tenían el diagnóstico de anomalías coronarias en el reporte, lo que podría haber incrementado la detección de algunas anomalías.

Respecto a las anomalías de origen desde el seno coronario opuesto, nuestra prevalencia (2,3%) fue un poco mayor a la presentada por Cheezum *et al*. ^6^, quienes encontraron 1,7%. Al igual que ellos, en nuestro estudio la principal arteria anómala fue la CD, así como el principal trayecto que siguió este grupo de anomalías fue el interarterial. La estenosis en el origen de la arteria anómala no fue significativa en la mayoría de los pacientes (estenosis menor a 50%), similar a lo encontrado por estos autores. Del grupo de anomalías de origen desde el seno coronario opuesto, las que cobran mayor importancia son las que presentan trayecto interarterial debido al riesgo de muerte súbita que pueden presentar los pacientes, en especial cuando la arteria de origen anómala es el TCI ^20^.

Entre las anomalías benignas de origen se encuentra el origen independiente de las arterias DA y CX desde el seno coronariano izquierdo, con agenesia de TCI, que tuvo una prevalencia de 0,27%, la cual está en el rango de prevalencia reportada en la literatura, donde varía de 0,112 a 0,48% ^16^^,^^17^^,^^21^. Otra anomalía usualmente benigna es el origen alto de una arteria coronaria, el cual se define como el nacimiento del ostium a 5 mm o más por encima de la unión sinotubular aórtica ^3^. En la literatura se reporta una prevalencia de 0,07 a 0,19 ^16^^,^^17^^,^^20^. La prevalencia encontrada fue de 0,67%, siendo mayor a lo reportado, y en todos los casos se trató de la CD, de ellos solamente un paciente presentó trayecto interarterial.

Respecto al origen de una arteria coronaria desde la arteria pulmonar, solamente encontramos origen anómalo del TCI (ALCAPA), con una prevalencia de 0,34%, la cual es una anomalía maligna, cuya prevalencia ha sido mayor que la encontrada en la mayoría de los estudios de imágenes (0,008 a 0,11) ^4^^,^^17^^,^^21^. Esta mayor prevalencia podría deberse a que esta anomalía se suele manifestar clínicamente a edades tempranas (todos los pacientes con ALCAPA fueron pediátricos). Sin embargo, nuestra prevalencia es menor si la comparamos con datos obtenidos en autopsias. En un estudio de autopsias de 1200 pacientes, la prevalencia de ALCAPA fue de 0,42% ^22^, debido a que es una de las anomalías coronarias que puede llegar a presentar muerte súbita, por lo que es más reportada por esta modalidad.

La arteria coronaria única se define como una sola arteria coronaria con nacimiento de la aorta de un único ostium y que brinda irrigación a todo el corazón, independientemente de su distribución ^23^. Encontramos una prevalencia de 0,27%. En la literatura, la prevalencia de arteria coronaria única oscila entre 0,044 a 0,23% ^13^^,^^21^^,^^24^^,^^25^. Dentro de este grupo se encuentra la ausencia congénita de CD, cuya prevalencia fue de 0,07% en nuestro estudio; en la literatura se reporta una prevalencia entre 0,011 a 0,017 ^16^^,^^21^^,^^24^.

Entre las anomalías de la anatomía arterial coronaria intrínseca predominó la doble DA, con una prevalencia de 0,47%. Siendo algo menor que lo reportado por ACI por Spíndola-Franco *et al.* ^(^^14^ (1%), mientras que en estudios por tomografía la prevalencia oscila entre 0,045 - 1,3% ^16^^,^^17^^,^^26^. La mayoría de casos en nuestro estudio fueron doble DA tipo I. Uno de los casos de doble DA tipo IV estuvo asociado a arteria coronaria única que se originaba del seno coronariano derecho, con TCI con trayecto retroaórtico (Tipo R II - P de Lipton) y que daba origen a la DA corta, mientras que la DA larga nacía de la arteria coronaria única con trayecto prepulmonar (Figuras 4E y 4F). Esta asociación de anomalías es raramente reportada, en la presente revisión se encontró un caso similar descrito en la literatura ^27^.

La doble CD se define como un sistema coronario derecho formado por dos ramas distintas que transcurren cercanas en el surco auriculoventricular, durante al menos la mitad de todo el trayecto de la CD, las dos ramas CD tienen diámetros similares. A veces pueden originarse en diferentes ostiums desde el seno coronariano derecho, y en otros casos, la CD se origina en un solo ostium y se divide en dos ramas después de una corta distancia variable del tronco proximal ^28^. La prevalencia de doble CD fue de 0,07% en nuestro estudio. Esta rara anomalía a veces no es registrada en los estudios de anomalías coronarias. Yamanaka *et al.*^21^ en su estudio de 126 595 ACI, no reportó esta anomalía. Kunimasa *et al.*^29^^)^ en su revisión de 2957 tomografías coronarias encontraron dos casos de doble CD, con una prevalencia similar a la nuestra (0,07%); mientras que Gräni *et al.*^17^ reportaron solo un caso en 5634 pacientes evaluados (prevalencia de 0,02%).

El origen ectópico de la primera rama septal desde un sitio distinto al de la DA es una anomalía coronaria rara poco reportada en la literatura. Algunos estudios de anomalías coronarias no lo describen ^4^^,^^15^^-^^18^^,^^21^. En esta anomalía, la primera rama septal puede originarse directamente de la aorta, de la porción proximal de la CD (como circulación colateral a la DA), desde el TCI, CX, primera rama diagonal o primera rama obtusa marginal ^30^^,^^31^. El origen ectópico de la primera rama septal desde el TCI tuvo una prevalencia de 0,07%. En el estudio de Verna *et al.*^31^, revisaron 700 ACI y encontraron una prevalencia de 1,1%, de los cuales solo un paciente presentó origen de la primera rama septal desde TCI (prevalencia de 0,14%).

Respecto a las anomalías coronarias de término, las fístulas de arterias coronarias se definen como la comunicación vascular anormal entre las arterias coronarias con las cámaras cardiacas o algún segmento de la circulación pulmonar o sistémica ^32^. En nuestro estudio, la prevalencia de fístulas coronarias fue del 0,54%. La prevalencia de esta anomalía por ACI es de 0,05 a 1% ^21^^,^^33^, siendo mayor cuando se evalúa por TC, encontrándose una prevalencia de hasta 0,9% ^34^.

El presente estudio tiene la limitación de ser un estudio retrospectivo, en el cual no se pudieron incluir a algunos pacientes debido a que no se encontraban las imágenes en el sistema RIS PACS (Picture Archiving and Communication System) de INCOR, ocasionando una reducción en la muestra.

En conclusión, la prevalencia de anomalías coronarias detectadas por TC de 64 detectores en un Instituto Nacional del Perú fue de 4,71%. La principal anomalía coronaria fue el origen de la arteria coronaria derecha desde el seno coronariano izquierdo con trayecto interarterial.
